# E-Knitted Textile with Polymer Optical Fibers for Friction and Pressure Monitoring in Socks

**DOI:** 10.3390/s19133011

**Published:** 2019-07-08

**Authors:** Claire Guignier, Brigitte Camillieri, Michel Schmid, René M. Rossi, Marie-Ange Bueno

**Affiliations:** 1 Laboratoire de Physique et Mécanique Textiles, Ecole Nationale Supérieure d’Ingénieurs Sud-Alsace, Université de Haute-Alsace, 11 rue Alfred Werner, F-68093 Mulhouse, France; 2 Empa, Swiss Federal Laboratories for Materials Science and Technology, Laboratory for Biomimetic Membranes and Textiles, Lerchenfeldstrasse 5, CH-9014 St. Gallen, Switzerland

**Keywords:** smart textiles, polymer optical fiber, pressure sensor, friction sensor, plantar pressure, gait analysis

## Abstract

The objective of this paper is to study the ability of polymer optical fiber (POF) to be inserted in a knitted fabric and to measure both pressure and friction when walking. Firstly, POF, marketed and in development, have been compared in terms of the required mechanical properties for the insertion of the fiber directly into a knitted fabric on an industrial scale, i.e. elongation, bending rigidity, and minimum bending radius before plastic deformation. Secondly, the chosen optical fiber was inserted inside several types of knitted fabric and was shown to be sensitive to friction and compression. The knitted structure with the highest sensitivity has been chosen for sock prototype manufacturing. Finally, a feasibility study with an instrumented sock showed that it is possible to detect the different phases of walking in terms of compression and friction.

## 1. Introduction

For several years, smart textiles have been a fast-growing development and there has been an increasing market for these materials. In these textiles, optical fibers can be integrated as sensors for various applications [1], for instance as monitoring of structures in civil engineering [2], seat occupancy in automotive industry [3,4,5], and position analysis [6,7]. However, these textiles are particularly interesting in the field of medical textiles. In the health and wellbeing domain, smart textiles are now used in various monitoring and therapy applications, making it possible to integrate the measurement of physiological parameters into daily life, or specific activities [8,9]. Furthermore, these textiles allow the taking of measurements at the surface of the skin without causing discomfort to the patient. The design of smart textiles for health and wellbeing monitoring is undergoing constant evolution and different strategies can be used: sensor yarn directly from the yarn constitutive material [10,11] or from a coating [12], sensor coating [13], or printing [14] on textiles. The sensor materials can be piezo-resistive [10,12,13,14,15,16,17], or piezoelectric [11,18,19]. The use of optical fibers for their light transmission and diffusion properties [20,21,22], or their sensor properties [23], offers new possibilities in the smart textile field, but this area has been less explored than the piezo-resistive effect.

The goal of this paper is not to compare the different sensing technologies but to prove the ability of POF to be inserted in a fabric, from an industrial machine, and to measure not just pressure, but also friction, when walking. Currently, by using optical fibers, it is possible to monitor various physiological parameters such as breathing rate [24,25,26,27,28], cardiac rate [24,29], pulse oximetry [30], and pressure [3,31,32,33]. Indeed, some polymer optical fibers (POFs) present several desirable properties such as a high bending ability, which can allow for easy integration within a textile [34,35]. Various studies have already shown that POFs can be integrated into textiles by weaving [3,32,36], sewing [25], and embroidery [30]. However, few studies have dealt with the integration of POFs into knitted structures [37,38], which is essential for near-to-body applications and to provide comfort. 

One of the most important points regarding the integration of POFs is that this must be possible, directly, during the manufacturing processing, in the same way as basic textile yarns, meaning that POF must support the constraints of textile processing. In this study, the focus is on the use of POFs as pressure and friction sensors, by integrating them from an industrial scale-up into a textile, and more precisely, into a knitted structure such as a sock to allow measurement of the pressure and friction on the feet/sock/shoe system. 

One possible application is the design of a monitoring sock that can detect conditions that are conducive to the development of pressure sores in diabetic foot pathology. The diabetic foot is a pathology with a prevalence of 6.4% worldwide [39], and which continues to increase. A knowledge of the pressure and friction under the foot is also essential in detecting favorable conditions for the development of a decubitus ulcer [40,41,42,43]. One approach consists of inserting sensors into a shoe insole, and the sensors may be optical fiber-based [44,45,46,47,48]. However, from a tribological point of view, it is better to take measurements of pressure and friction, as far as possible, at the investigating contact, i.e. the surface of the skin, or within the sock, rather than at an area further away, such as on the shoe sole, for example. Some smart socks have been reported in the literature or are on the market. They can measure the pressure on the foot at several positions within the sock, localized on specific areas of the sole, using piezo-resistive yarns [49,50,51,52] patches made from yarn [53] or polymer plate [54,55]. Other proposed methods include socks sensitive on the whole surface with a specific sensor yarn integrated in the socks, such as Alpha-Fit [56], or made from a piezo-resistive complex of three layers of 2D fabrics sewn to obtain a foot shape and adapted only for prosthesis [57], or the forming of a sensor sewn on the sock [58].

With weft knitting technology, it is possible to directly produce shaped 3D knits with the inlay technology that is commonly used for compression stockings, allowing the reinforcement of knits with precise control over the position of the inlay yarn inside the structure. The aim of this study was to determine the properties of different POFs in terms of the constraints applied during knitting, in order to choose the best POF for knitting. The chosen POF was then knitted into 2D fabrics and its behavior was analyzed under friction and compression. Finally, optical fibers were knitted directly into a sock, and a feasibility study of the detection of compression and friction during walking was performed.

## 2. Materials and Methods

### 2.1. POF Implementation in Knitted Structures

**Figure 1:**
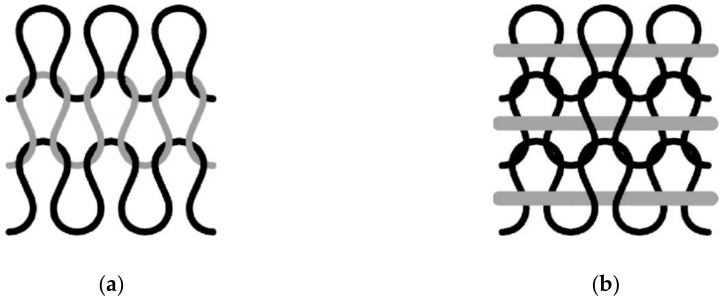
(**a**) Example of a knitted structure with loops (single jersey); and (**b**) the same knitted structure with an inlay yarn (the ground yarn making loops is shown in black and the inlay yarn in grey).

A sock is a 3D shape made from loops obtained by knitting. A knitted fabric is made up of interconnected loops made from a yarn (Figure 1a). During the knitting process, the yarn is stressed in bending, traction and friction, against metallic parts of the machine [59,60,61]. In the knitted fabric, the yarn is drastically bent to form a loop. During the process, the curvature, subjected on the yarn, depends on the stitch length (in mm/stitch) and the needle thickness. However, an important curvature has to be avoided with an optical fiber, because of a loss of the light signal at the output of the fiber. For the intended application, the POF had to be inserted into the fabric in such a way as to avoid curvature, meaning that it could not be directly knitted, forming a loop, but had to be as straight as possible within the fabric structure. The chosen solution was to insert an inlay yarn inside the structure (Figure 1b). This solution is commonly used for elastic yarn in the socks ribbing part, for example.

Another advantage of inlay is that during the process, tension and friction are acting on the inlay yarn to a lesser extent than on the ground yarn. 

However, even if the inlay yarn is straight in the structure, when in use, the knitted fabric is handled and stressed in tension and bending, corresponding, essentially, to bending of the yarn, sometimes under critical curvature. Therefore, the POF has to be flexible, i.e. to have a low bending rigidity *B*, which depends on the Young’s modulus *E* and the moment of inertia of the cross-section *I*, and which, for a circular fiber, is:

(1) $$B=\;E\cdot I=E\cdot \frac{\pi \cdot d^{4}}{64}$$

where *d* is the diameter of the fiber.

Moreover, the POF has to be elastic, i.e., recover its integrity after bending. 

Therefore, this work focused on two kinds of characterization: tensile properties and bending properties, even if the required properties of the yarn in the industrial process are not quantified and are instead empirically determined by testing on a knitting machine. 

Tensile tests were carried out using an MTS tensile machine with a specific fixation system to avoid sliding and breaking at the grippers. A 2 kN sensor was used, and the tests were carried out with a speed of 500 mm/min.

**Figure 2:**
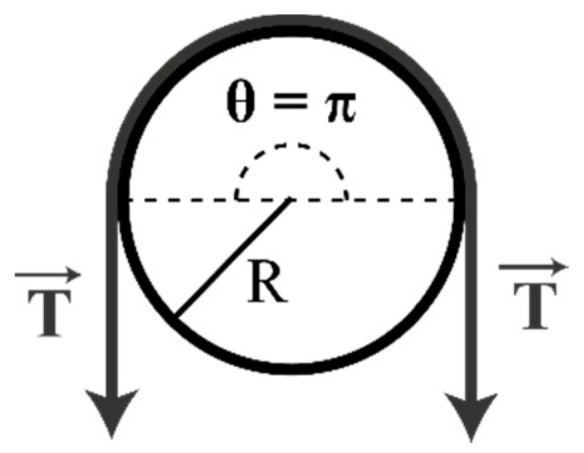
Principle of the bending behavior test.

The bending properties of the fibers were then investigated. These tests were performed with an optical fiber, 30 cm in length, with various radii of curvature (R varied from 0.5 mm to 2.5 mm). An axial tension of 1.6 cN/tex was applied on the yarn (where a tex is a unit corresponding to the weight in grams of 1 km of yarn), as shown in Figure 2. This corresponds to the normal tension applied on the yarn during the knitting process. 

For the ground yarn made of classical textile fiber, three basic knitted structures were chosen: a single jersey, a 1 × 1 rib and a 1 × 1 interlock. The single jersey was thinner than the other two fabrics, and the 1 × 1 interlock was denser than the 1 × 1 rib. Single jersey is composed only of a single side (with face stitches), while 1 × 1 rib and 1 × 1 interlock have two sides (face and reverse stitches). The optical fiber was inserted into the knitted structure by wedging it between the wales of the stitches (Figure 1b).

These structures were made using an industrial flat knitting machine CMS multi gauge ADF 32 BW from Stoll AG & Co. (Reutlingen, Germany). Although socks are typically made on dedicated hosiery machines, the advantage of the machine used here was its versatility and thus the ability to knit all kinds of 3D shapes, for example socks, but also complex 3D shapes in the same way as a real additive manufacturing machine.

### 2.2. Polymer Optical Fibers

Optical fibers, produced with different polymers, were analyzed in order to determine which was most suitable to be integrated into a textile structure. Because of the need to have flexible POFs, polycarbonate (PC) and polymethylmethacrylate (PMMA) fibers have been avoided. Three different POFs have been investigated:

- GigaPOF 50-SR from Chromis Fiberoptics (Warren, NJ, USA), made with perfluorinated polymer CYTOP^®^ [62], with a core diameter of 50 µm and a cladding of 490 µm. According to the manufacturer, this POF supports a maximum tensile load of 7 N and has a minimum bending radius of 5 mm.
- A bi-component fiber made of cyclo-olefin/fluorinated polymer and provided by Empa (ref. 1144), with a diameter of 144 ± 4 µm.
- A mono-component fiber using a Geniomer^®^ 100 polymer, with a diameter of 824 ± 13µm, also produced by Empa (ref.1263).

Previous studies have described the various properties of these fibers, for GigaPOF [3,4,5,35] and for Empa POFs [30,63,64].

### 2.3. Friction and Compression Measurements

**Figure 3:**
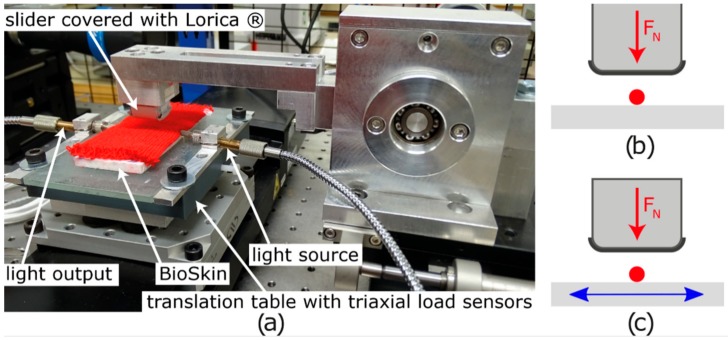
(**a**) Picture of the measurement system and schematization of the testing configuration for (**b**) compression tests and (**c**) friction tests.

Friction and compression were measured using a tribometer designed for friction measurement and adapted to measure transmitted light. This tribometer was composed of a translation table (model M-ILS100CC, Newport Corporate, Irvine, CA, USA) in which a triaxial load cell (model 3A60-20N, Interface Inc., Scottsdale, AZ, USA) was integrated, in order to measure the normal load and the tangential load during the test. The normal load was applied with a fixed arm, and its vertical position was modified to apply the desired normal load. The system is represented in Figure 3a. In this study, the slider has a rectangular sole with an area of 1.5 cm^2^ (1.5 cm length and 1.0 cm width) and is covered with Lorica^®^, which has frictional properties close to those of the human skin [65]. Furthermore, an artificial skin (polyurethane elastomer BIOSKIN with rough surface, Beaulax Co., Saitama, Japan) was placed underneath the knit to give the same hardness as real skin. 

The compression tests consisted of applying a cyclic normal load to the optical fiber (Figure 3b). Friction tests were realized using a normal load on the optical fiber, with a sliding distance of 30 mm perpendicular to the POF, and a sliding speed of 20 mm/s. Five cycles were carried out for each measurement (see Figure 3c).

Friction and compression tests were performed under four normal loads, 0.45, 3, 10, and 18 N, in order to give a pressure between 3 kPa (touch pressure) and 120 kPa (mean of the dynamic pressure during walking) [43,66]. In order to obtain repeatable and reproducible results, measurement was performed on five samples for each normal load and was repeated five times for each sample. Each test also involved five compression/friction cycles, meaning that for each normal load and each configuration, 125 measurements were performed.

### 2.4. Light Measurement

A light was added to the friction and compression setup in order to connect the optical fibers to a halogen white light source (Avalight HAL 10 W, Avantes, Apeldoorn, Netherlands). The optical fibers were coupled by means of SMA connectors (Thorlabs Inc., Newton, NJ, USA). 

The light measurement relied on the light transmission through the optical fiber, and the irradiance was recorded and converted into voltage by a photodiode (model 918D-SL-OD3R, Newport Corporate, Irvine, CA, USA). The photodiode signal was connected to a Pulse data recorder (Brüel & Kjaer, Mennecy, France).

The light source and the coupling stability have been investigated in previous work, and it has been shown that they can be considered constant [67]. Hence, the transmission of the light only depends on the fiber deformation due to pressure and friction. Because the fiber does not have exactly the same path from one sample to another, the absolute irradiance is not the same. Therefore, the relative irradiance will be considered, and more precisely, the irradiance loss for all the results:

(2) $$Irradiance\;loss\left(i\right)=\;\frac{Irrad_{max}-Irrad_{i}}{Irrad_{max}}$$

*Irrad_i_*: instantaneous irradiance; *Irrad_max_*: maximal irradiance of the fiber during test (without applied load).

The irradiance loss varies from zero (no loss of light, i.e. when no stress is applied to the POF) to 100% (when no light is transmitted through the POF due to the applied stresses).

## 3. Results

### 3.1. 2D Knitted Fabrics with Insertion of Optical Fibers

#### 3.1.1. Choice of Polymer Optical Fiber

In order to determine the POF that is best suited to integration into a knitted structure, as explained in Section 2.1, the bending rigidity and recovery of the POFs after bending have been evaluated.

**Figure 4:**
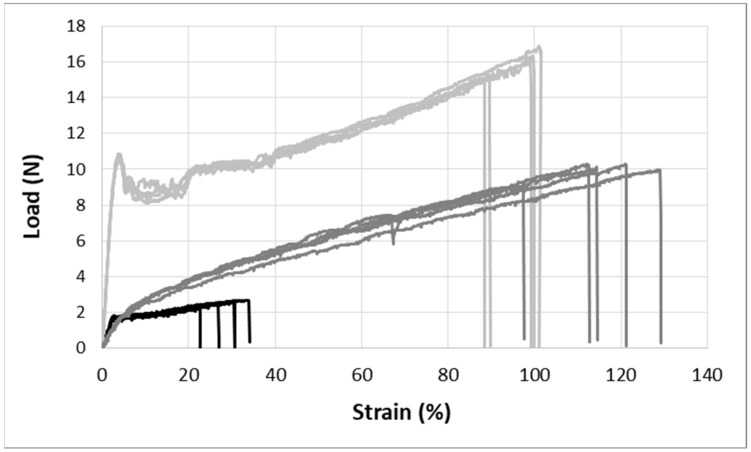
Load-strain curve for the three studied optical fibers: Empa bi-component (black), Empa Geniomer^®^ (dark grey) and GigaPOF 50-SR (soft grey).

Figure 4 presents the load-strain curves obtained for the three POFs. It can be clearly seen that the bi-component POF has the lowest tensile properties. The GigaPOF 50-SR and the Geniomer^®^ POF have a large elongation at breaking, while the core of the GigaPOF 50-SR fiber broke at the beginning of the tensile test, i.e. around 5% of elongation, although the cladding showed higher tensile properties and did not break immediately. Hence, the curves presented here over-evaluate the real loading at breaking for this fiber. The Geniomer^®^ POF allowed a high level of elongation, which is compatible with textile applications. From these force-elongation curves, and the fiber section, the Young’s modulus of each POF has been calculated (Table 1). It can be observed that despite its high diameter, the Empa Geniomer^®^ POF is the least rigid. 

The bending rigidity (Equation (1)) has been calculated (Table 1), and the most flexible is the Empa Geniomer^®^ POF.

**Figure 5:**
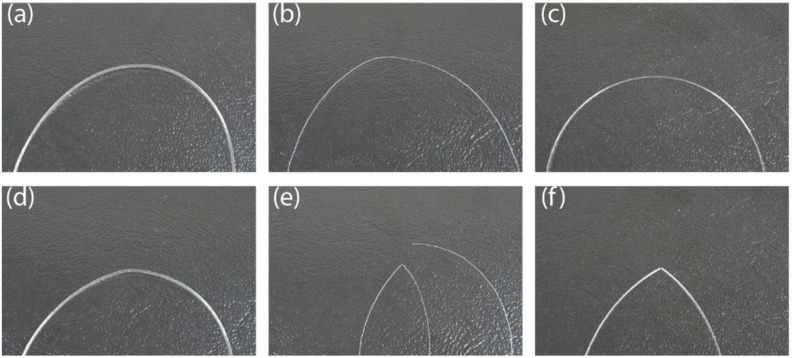
Evaluation of the plastic behavior of optical fibers under bending, with a radius of 1 mm: Empa Geniomer^®^ (**a**) before testing and (**d**) 2 min after testing; Empa bi-component (**b**) before testing and (**e**) 2 min after testing; GigaPOF 50-SR (**c**) before testing and (**f**) 2 min after testing.

For the bending recovery test (Figure 2), the curvature under tension was applied to the fiber for 30 s then the load was removed. A picture of the fiber was taken just after the removal of the tension and again after 2 min. Figure 5 shows the aspect of the fibers immediately after removal of the tension and after 2 min, for a radius of 1mm. 

The results show that the GigaPOF 50-SR and the bi-component fibers demonstrated plastic behavior for bending radii smaller than 5 mm and 4 mm, respectively. However, the mono-component fiber from Empa, with Geniomer^®^, underwent elastic deformation, even for a bending radius of 1 mm. 

For this reason, Geniomer^®^ POF was chosen as the best candidate for industrial insertion into a knitted structure, as it showed the best bending properties. 

#### 3.1.2. 2D Knitted Fabrics

**Figure 6:**
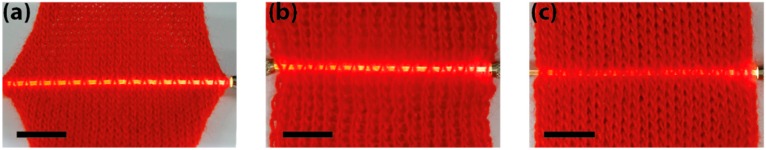
Pictures of the knitted fabrics: (**a**) single jersey; (**b**) 1 × 1 rib; and (**c**) 1 × 1 interlock fabrics. The scale bar indicates 10 mm.

Insertion of the optical fiber into the knitted structure was realized using the inlay technique for three knitted structures, as described in Section 2.1. Figure 6 presents the obtained knits. Five samples were produced for each type of fabric. The stitch lengths (the length of the ground yarn in a single loop, in cm/stitch) were measured according to the NF EN 14970 standard. The results were 0.74 ± 0.01 cm/stitch for the single jersey, 0.89 ± 0.01 cm/stitch for the 1 × 1 rib, and 0.88 ± 0.01 cm/stitch for the 1 × 1 interlock. Figure 6a highlights the curling effect of the single jersey, which was not shown by the other knitted fabrics (Figure 6b,c).

### 3.2. Knitted Fabric Compression Sensitivity

The goal of this section is to determine the best structure for compression measurement.

#### 3.2.1. Measurement Repeatability and Reproducibility

In order to determine the repeatability of the measurement setup, five consecutive compression cycles were made for each sample under the same testing conditions. The five measurements were made from the same sample. 

**Figure 7:**
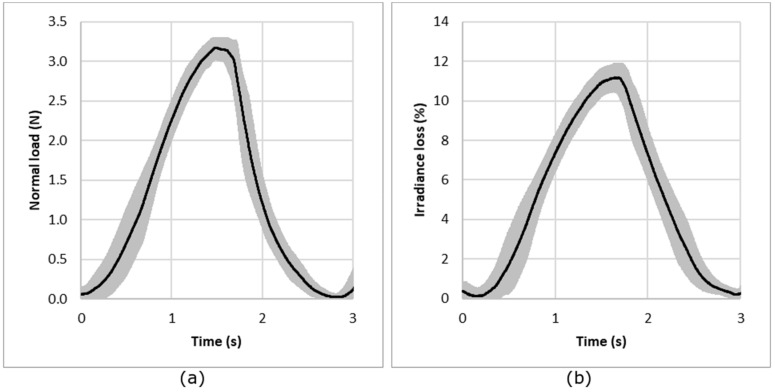
Repeatability of the measurement: evolution of the compression load (**a**) and irradiance loss (**b**) for a compression test (example with single jersey under a nominal load of 3 N). The black line represents the average curve and the gray area the standard deviation.

For repeatability evaluation, the procedure consists of measuring normal load and irradiance loss (Equation (2)) for a sample under compression. The sample was removed and replaced after each measurement. The experience has been done for a sample per structure and for each normal load. Figure 7 presents the results for the knitted jersey fabric under compression conditions of 3 N load. The results show that the repeatability of the measurement is good, with a mean CV% of the maximum irradiance loss from all the tests calculated from the CV% for each normal force; each knitted structure and each sample is less than 8%.

**Figure 8:**
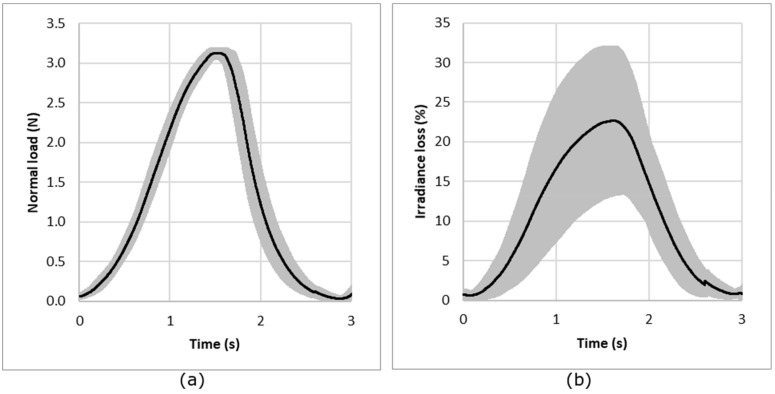
Reproducibility of the measurement: evolution of the compression load (**a**) and irradiance (**b**) for a compression test (example with single jersey under a nominal load of 3 N). The black line represents the average curve and the gray area the standard deviation.

In another way, the reproducibility of the measurements and particularly the behavior of the optical fiber was investigated using measurements of five different samples with the same structure. This test was done for each structure and each normal load. Figure 8 presents the results for compression loading and irradiance loss obtained for the jersey knitted structure for a load of 3 N. The results show the reproducibility from one sample to another. The results obtained show a prior calibration of the POF will be needed for a commercial use. In fact, the mean CV% of the maximum irradiance loss from all the tests calculated from the CV% for each normal force and each knitted structure is 36%.

This defect in reproducibility is due to a non-perfectly flat POF cross section and a non-perfect connection, possibly due to an irregularity of the POF.

#### 3.2.2. Influence of the Knitted Fabric Structure

In order to determine the best structure, the sensitivity of the whole sensor, i.e., knitted fabric with inlaid POF, was determined during compression.

**Figure 9:**
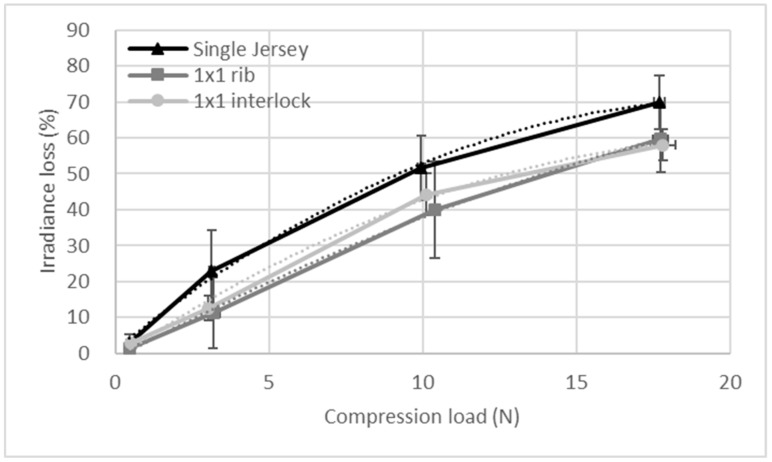
Evolution of the loss of irradiance relative to the applied compression load for the three knitted structures with the error bars representing the standard deviation. The dotted lines correspond to the second order fitting curves.

Figure 9 shows the evolution of the mean loss of irradiance with respect to the compression load applied on the optical fiber for the three knitted structures investigated. Each mark corresponds to 125 measurements (five cycles per measurement, five measurements per sample, five samples per structure). The curves can be fitted by a second-order polynomial with a coefficient of determination higher than 0.99 (Table 2). 

The evolution of the irradiance loss, relative to the compression load, shows a polynomial evolution, and there is correlation of the irradiance loss with the compression load, i.e., the fiber deformation. The single jersey presents the highest values of irradiance loss, and therefore the highest sensitivity. This may be due to the fact that this structure was the thinnest. The optical fibers in this fabric are more stressed in compression than the optical fibers within the 1 × 1 rib and 1 × 1 interlock, since in these structures the optical fiber is more protected because of the number of sides (see Section 2.1) 

### 3.3. Knitted Fabric Friction Sensitivity

#### 3.3.1. Measurement Repeatability and Reproducibility

The repeatability and reproducibility of the measurements was investigated using the same sample under the same testing conditions as used in Section 3.2.1 for compression. Due to the results obtained from the compression test, the results are expressed in terms of irradiance loss.

**Figure 10:**
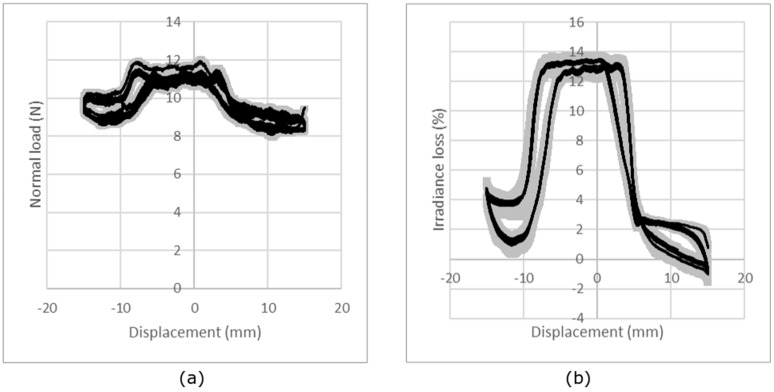
Repeatability of the measurements: (**a**) evolution of the normal force and (**b**) evolution of the loss of irradiance relative to the displacement of the slider for five repetitions of the measurement on the same single jersey sample under a nominal load of 10 N. The black line represents the average curve and the gray area the standard deviation.

The repeatability of the measurement is illustrated in Figure 10, which, as an example, presents the results obtained for the single jersey knitted fabric, corresponding to five measurements on the same sample. It can be observed that the repeatability is good, with a mean CV% of the maximum irradiance loss from all the tests, calculated from the CV% for each normal force, each knitted structure and each sample, is 20%. 

**Figure 11:**
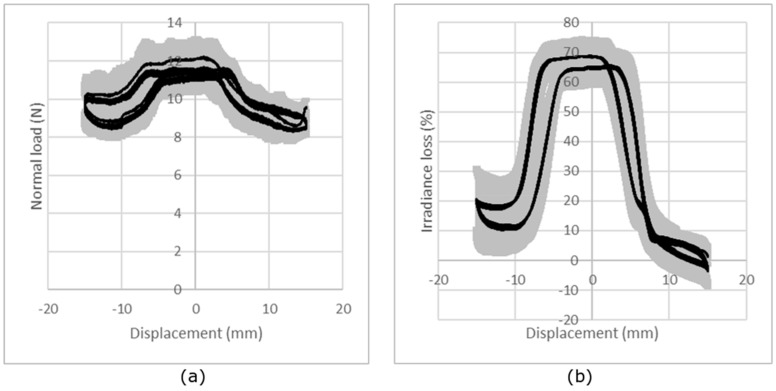
Reproducibility of measurements: (**a**) evolution of the normal force and (**b**) evolution of the loss of irradiance relative to the displacement of the slider for five samples of single jersey under a nominal load of 10 N. The black line represents the average curve and the gray area the standard deviation.

The reproducibility of the measurements and the behavior of the optical fibers were investigated for five samples and are illustrated in Figure 11, which presents the results obtained for the single jersey knitted fabric under friction conditions of a 10 N load. The two parts of the normal force and irradiance loss signals, representing the forward and backward cycles, are shifted due to the movement of the optical fiber inside the structure and the fact that the knit is not glued to the BIOSKIN. This shift can also be observed in the irradiance loss. It can be concluded that the reproducibility obtained for five different samples is quite good. In fact, the mean CV% of the maximum irradiance loss from all the tests, calculated from the CV% for each normal force and each knitted structure, is 44%.

#### 3.3.2. Influence of the Knitted Fabric Structure

**Figure 12:**
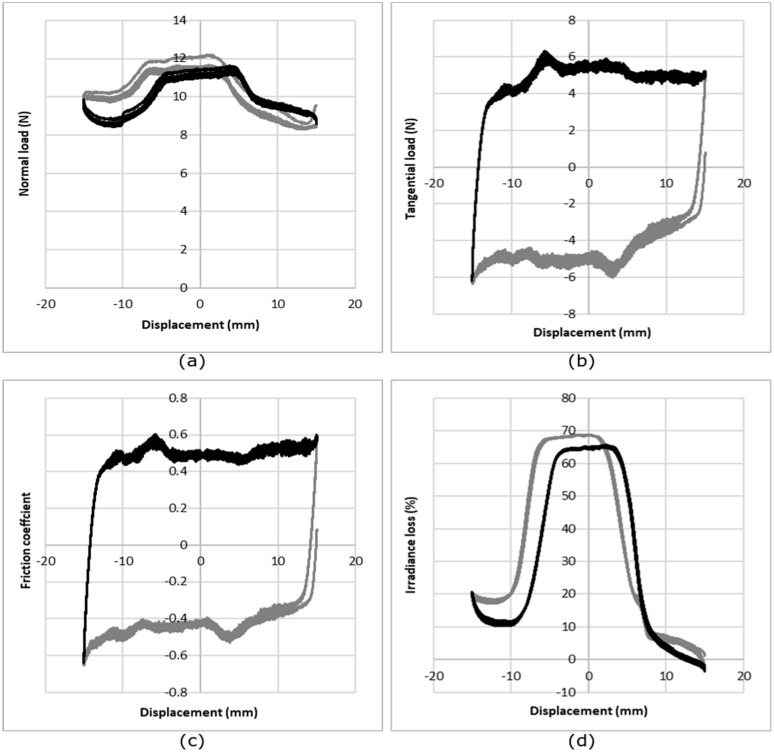
Evolution of (**a**) the normal load, (**b**) tangential load, (**c**) coefficient of friction, and (**d**) the loss of irradiance relative to displacement for the single jersey knit under a nominal load of 10 N. The grey line shows the forward and the black the backward cycle of the slider. Each curve corresponds to the average of 25 measurements.

The sensitivity of the three instrumented textile fabrics has been tested in friction. Figure 12 shows the raw signal of the normal load (Figure 12a), tangential load (Figure 12b), coefficient of friction computed from the two previous curves (Figure 12c), and the evolution of the irradiance loss (Figure 12d), relative to the displacement. It can be seen that the normal load gives a higher value for certain displacement values, corresponding to the moment when the slider is located above the optical fiber. The increase in the irradiance loss occurs when the normal load is the highest, indicating that it is due to the friction of the slider on the optical fibers. Based on these results, the means of the normal load, tangential load and irradiance loss were calculated for the optical fiber displacement due to friction, using the 125 measurements for each condition (five cycles per measurement, five measurements per sample, five samples per structure).

**Figure 13:**
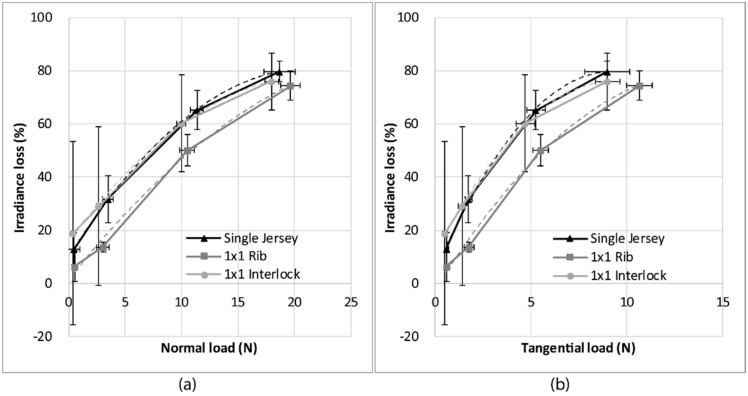
Evolution of the irradiance loss relative to (**a**) the normal load applied and (**b**) the tangential load for the three knitted fabrics, with the error bars representing the standard deviation.

Figure 13 shows the evolution of the irradiance loss relative to the normal applied load (Figure 13a) and the evolution of the irradiance loss relative to the tangential load (Figure 13b). The experimental curves were fitted with a second order polynomial trend line, and the equations of the fitting curve are given in Table 2.

The irradiance loss increases with both the normal load and the tangential load. In this case, the single jersey and the 1 × 1 interlock structures show similar behavior, and the 1 × 1 rib structure shows the smallest irradiance loss and the least sensitivity. Therefore, from the compression and friction tests, it appears that the structure giving the highest sensitivity is the single jersey. Therefore, this structure was chosen for the sock.

**Figure 14:**
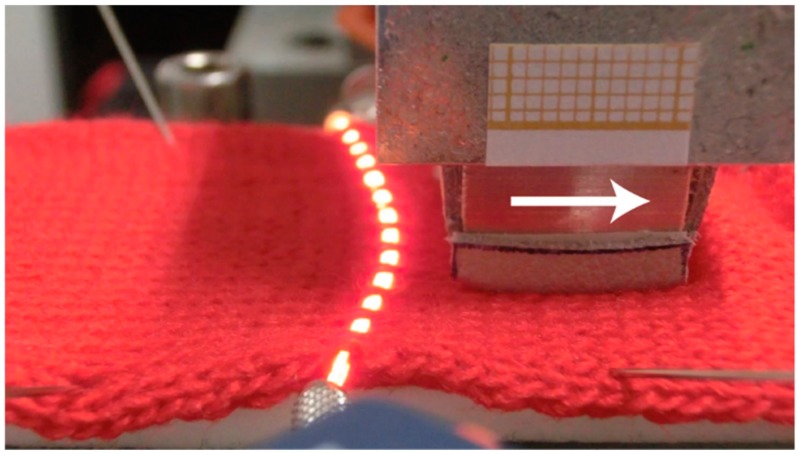
Deflection of the POF at the back of the slider in the single jersey fabric. The direction of movement is indicated by the arrow.

During the friction tests, several phenomena occur in the optical fibers, arising from a combination of compression and friction due to the movement of the slider. Since the knits are not glued to the BIOSKIN, the optical fiber tends to move during the tests and to bend at the surface of the fabric (Figure 14). This also modifies the path of the light inside the optical fiber and has an impact on the irradiance loss. At the scale of the knitted structure, this phenomenon can be explained by the fact that the single jersey and 1 × 1 interlock fabrics have structures in which the optical fibers are more supported, meaning that their movement inside the structure is less significant. When the fabric moves, the optical fiber follows the movement of the knit, causing a large deflection of the POF and higher irradiance loss. 

**Figure 15:**
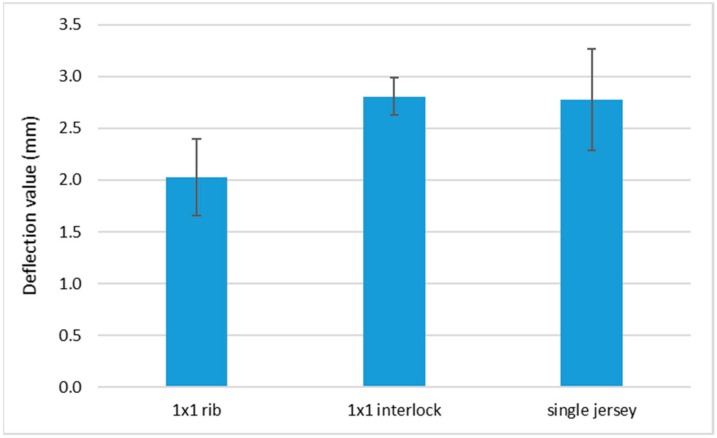
Deflection values of the POF at the back of the slider for the three fabrics.

Conversely, for the 1 × 1 rib, which has fewer interlacing points between two consecutive courses, the optical fiber is less supported inside the structure, and the optical fiber moves less than the knit during the friction. This explanation is confirmed by the deflection value of the POF at the back of the slider for the different structures (Figure 15). For the 1 × 1 rib, the deflection is lower than for the 1 × 1 interlock and single jersey structures.

In conclusion, from the compression and friction tests, it appears that the structure giving the highest sensitivity is the single jersey. Therefore, this structure was chosen for the sock.

### 3.4. Knitted Fabric Friction Force Sensitivity

During the compression and friction tests, a normal load is applied on the fabric, but only in the friction test was a tangential force also applied. The goal of this section is to determine if the knitted fabric, with the implemented POF, is sensitive to normal force and to friction force, i.e., tangential force. The three knitted structures are used in this section.

**Figure 16:**
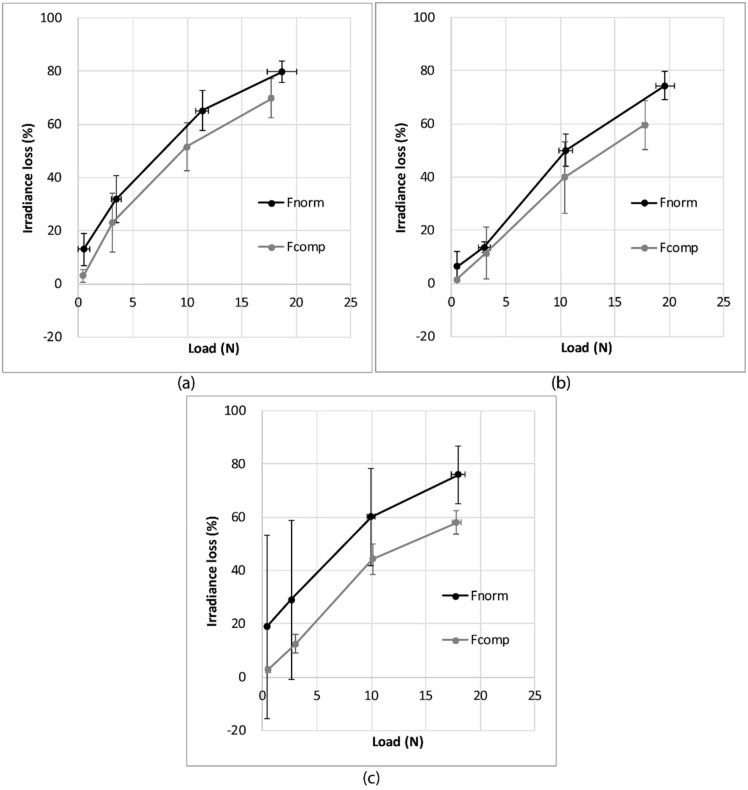
Comparison between the evolution of the irradiance loss as a function of the normal load during friction (F_norm_) or compression tests (F_comp_) for (**a**) single jersey; (**b**) 1 × 1 rib; (**c**) 1 × 1 interlock, with the error bars representing the standard deviation.

Figure 16 presents a comparison of the irradiance loss obtained in the compression tests and the friction tests for the three knitted fabrics. It can be shown that under a given normal load, the irradiance loss is always higher in the friction tests than in the compression tests for all three structures. 

It can therefore be concluded that the optical fiber is more sensitive to friction, including normal and tangential load, than compression under the same normal load. 

## 4. Proof-of-Concept of the e-Knitted Textile with POF for Walking Monitoring

**Figure 17:**
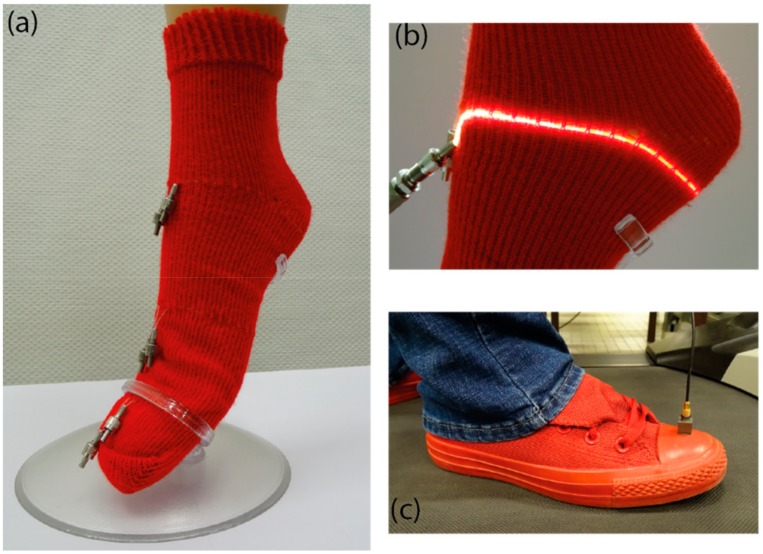
(**a**) Picture of the knitted sock with the three optical fibers; (**b**) magnification of the optical fiber at the heel position; (**c**) setting up the accelerometer.

A sock, with the Empa Geniomer^®^ POF inlaid in a single jersey ground, has been knitted. In order to determine whether the chosen optical fibers were able to measure the pressure and friction during walking, several experiments were performed after the insertion of three optical fibers at three different positions inside the sock: heel, mid-foot and metatarsal areas (Figure 17). These zones correspond to the foot pressure zones [42,43,68].

**Figure 18:**
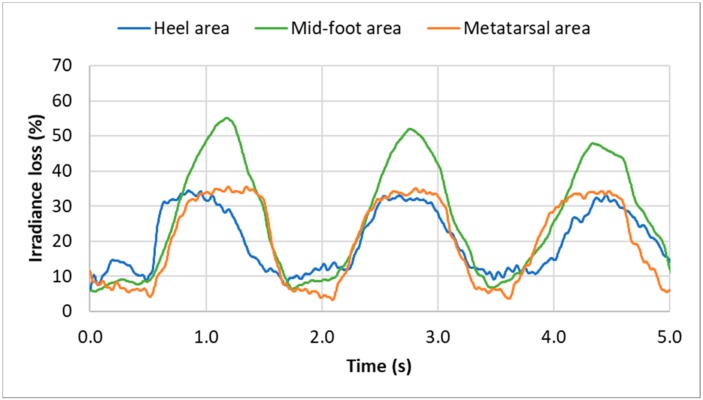
Evolution of the irradiance loss during walking at 2 km/h for three optical fibers placed at three different positions under the feet, for three walking steps. Each curve is the average of five measurements.

Figure 18 shows the evolution of the light signal from the three POFs in the different areas and for several walking steps. It can be clearly seen that these results are reproducible.

**Figure 19:**
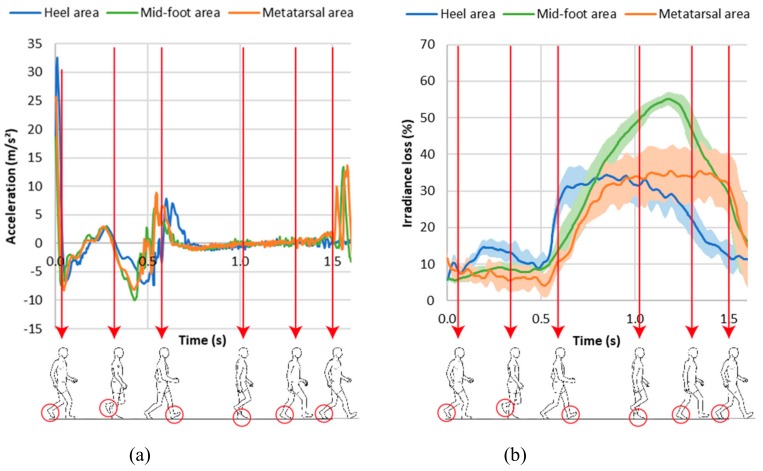
Evolution as a function of time of (**a**) the acceleration signals and (**b**) the light signals during walking at 2 km/h for three optical fibers placed at different positions under the foot and the corresponding step of the walking gait. Each curve is the average of five measurements. The plain lines represent the average curves and the gray areas the standard deviation.

In order to identify the different phases of the walk, an accelerometer was put on the shoe (Figure 17c) to synchronize with the POF’s signals. The starting point was determined with a video to correspond with the moment when the foot leaves the ground (Figure 19a). This point is used as a trigger for the signals from the three POFs. Figure 19b presents the results for the irradiance loss evolution (0% corresponds to the maximum irradiance during the gait). 

Under the present conditions, i.e. mean velocity and step length, the duration of the walking cycle was approximately 1.7 s (Figure 19a,b).

From Figure 19b, it can clearly be seen that the three optical fibers measure the different values for the light during walking. This means that the optical fibers inserted in the sock are sensitive to walking, and that the irradiance loss is highest when weight is applied to the optical fibers. Moreover, the three optical fibers show different types of light evolutions, meaning that it is possible to differentiate the fiber position from the irradiance signal. The heel area is first to come into contact with the ground, then the mid-foot area is in contact with the ground over a similar period to that of the metatarsal area. The heel and metatarsal areas are in continuous and constant contact around 0.5 s, while the mid-foot shows a maximum loss at 1.2 s after the beginning of the walking gait. This phenomenon may be explained by two mechanisms:

- In the mid-foot area, the real contact area is small, i.e. the external lateral area of the foot (isthmus),
- The change in the mid-foot cross-sectional shape induces a change in the global curvature of the POF. The mid-foot area is formed by softer tissues than the heel and metatarsal areas, and the sectional shape of the mid-foot changes during walking, which causes a change in the optical fibers leading to a modification of the light transmission.

These results confirm the feasibility of friction and pressure monitoring, allowing the evolution of these parameters to be studied during walking.

This experiment proves the measurement of friction and compression during walking is possible from a sock instrumented with a POF. However, this study has been done with a single participant, therefore, further investigations, with a greater number of volunteers, have to be arranged to really appreciate the limit of this configuration. 

## 5. Conclusions

The aim of this study was to determine the necessary properties of optical fibers to be inserted inside a knitted structure and to choose a fiber based on these properties. The chosen POF is the Geniomer^®^ 100 made by Empa with a 0.8 mm diameter, which will be reduced in the future. The optical fibers were inserted by inlay inside three knitted fabrics, and the behavior under compression and friction of the optical fibers was analyzed.

It was shown that the POF inside the knitted fabric is sensitive to both compression and friction. The sensitivity is higher under friction tests, when normal and tangential loads are combined. The chosen knitted structure is the single jersey which gives the fabric with the highest sensitivity.

Finally, a feasibility study regarding the behavior of optical fibers during walking was carried out. For this, three optical fibers were inserted inside a sock in three different zones. The results show that the optical fibers allow a walking gait to be maintained and measure stresses acting on the fibers in different zones.

## Figures and Tables

**Table 1:** Young’s moduli and bending rigidity of the POFs used. *POF provided by the Empa.

Optical Fiber Type	Young’s Modulus (GPa)	Bending Rigidity (10^−9^ N·m^2^)
GigaPOF 50SR	2.1 ± 0.1	44 ± 3
Bi-component POF*	4.7 ± 0.4	96 ± 18
Geniomer^®^ POF*	0.10 ± 0.02	2.0 ± 0.4

**Table 2:** Coefficients of the second order polynomial $y=ax^{2}+bx+c$ for compression (compression load) and friction tests (normal and tangential loads) for the three knitted structures studied with all the curves *R^2^* > 0.99.

Knitted Structure	Normal Force During Compression Test (N)	Normal Force During Friction Test (N)	Tangential Force During Friction Test (N)
Single jersey	a=−0.17;b=6.88; c=1.10	a=−0.16;b=6.67; c=10.07	a=−0.88;b=16.25; c=4.92
1 × 1 rib	a=−0.06;b=4.60; c=−1.57	a=−0.08;b=5.36; c=1.43	a=−0.41;b=11.58; c=−2.39
1 × 1 interlock	a=−0.14;b=5.77; c=−1.48	a=−0.13;b=5.67; c=15.98	a=−0.73;b=13.67; c=11.95
